# The application of global sensitivity analysis in the development of a physiologically based pharmacokinetic model for *m*-xylene and ethanol co-exposure in humans

**DOI:** 10.3389/fphar.2015.00135

**Published:** 2015-06-30

**Authors:** George D. Loizou, Kevin McNally, Kate Jones, John Cocker

**Affiliations:** Computational Toxicology Team, Mathematical Sciences Unit, Health and Safety LaboratoryBuxton, UK

**Keywords:** PBPK modeling, xylene and ethanol coexposure, human volunteer study, global sensitivity analysis, kinetics

## Abstract

Global sensitivity analysis (SA) was used during the development phase of a binary chemical physiologically based pharmacokinetic (PBPK) model used for the analysis of *m*-xylene and ethanol co-exposure in humans. SA was used to identify those parameters which had the most significant impact on variability of venous blood and exhaled *m*-xylene and urinary excretion of the major metabolite of *m*-xylene metabolism, 3-methyl hippuric acid. This analysis informed the selection of parameters for estimation/calibration by fitting to measured biological monitoring (BM) data in a Bayesian framework using Markov chain Monte Carlo (MCMC) simulation. Data generated in controlled human studies were shown to be useful for investigating the structure and quantitative outputs of PBPK models as well as the biological plausibility and variability of parameters for which measured values were not available. This approach ensured that *a priori* knowledge in the form of prior distributions was ascribed only to those parameters that were identified as having the greatest impact on variability. This is an efficient approach which helps reduce computational cost.

## Introduction

Exposures to chemicals in occupational and non-occupational settings have been linked to an increased incidence of disease (Rappaport, 2012; Rappaport et al., 2014). Understanding the relationship between exposure and disease typically requires a human health risk assessment (RA; Sahmel et al., 2010). Ideally, such an assessment must include translation of ambient exposure concentration to a relevant, biologically effective dose that may, in turn, be related to response (Bessems and Geraets, 2013; Geraets et al., 2014). Physiologically based pharmacokinetic (PBPK) models have been used successfully for many years to translate inhalation, dermal and oral exposure to single environmental chemicals and pharmaceuticals to ‘tissue dosimetry’ or concentrations within the organs and tissues (Clewell, 1995, 2005; Willhite and Clewell, 1997; Rostami-Hodjegan and Tucker, 2007; Jamei et al., 2009; Polak et al., 2012).

In reality, people are exposed to mixtures of chemicals present in the environment with the possibility that components may interact. Interacting chemicals can lead to lower toxicity (antagonism) or greater toxicity (synergism, potentiation) than that expected based on the potency and dose of the individual chemicals (Krishnan et al., 2002). Pharmacokinetic interactions can lead to a change in tissue dose of chemicals during exposure to a mixture compared with single exposures (Krishnan and Brodeur, 1994). Interestingly, Krishnan et al. (2002) demonstrated that with the application of PBPK modeling the extrapolation of binary interactions to more complex mixtures is possible and that this may afford a basis for a quantitative chemical mixture RA methodology.

PBPK models are independent, structural models, comprising compartments that correspond directly and realistically to the organs and tissues of the body connected by the cardiovascular system (Clewell and Andersen, 1985, 1987, 1994, 1996; Rowland et al., 2004; NRC, 2007; Zhao et al., 2011). The principle application of PBPK models is in the prediction of the appropriate form of the target tissue dose, or *dose-metric*, of the parent chemical or its reactive metabolite(s). The dose-metric must capture the critical biological steps that lead to an effect. Such mechanisms may take place within any compartment, e.g., blood, organ, or sub-cellular compartment. Use of an appropriate dose-metric in chemical RA calculations provides a better basis for relating to the observed toxic effects than the external or administered exposure concentration of the parent chemical (Conolly and Butterworth, 1995; Barton et al., 1998; IGHRC, 1999; Johanson et al., 1999; Andersen, 2003; Clewell and Clewell, 2008; Lipscomb and Poet, 2008; McNally et al., 2011). Therefore, PBPK models contain knowledge of the system being studied in the form of dozens of parameters and inputs that are associated with uncertainties. Yet, an aspect of the use of PBPK models, which continues to require study, is whether any particular model is an adequate representation of the biological system it is built to emulate. If there are inadequacies and uncertainties in the PBPK model then the tissue dosimetry estimates will be wrong. By using data generated from laboratory studies where both the biological monitoring (BM) outputs and the originating exposure (input) are known, the PBPK model structure can be evaluated and any inadequacies addressed. However, in the environmental and occupational toxicology arena there have been a limited number of validations of human PBPK models with BM data obtained in controlled human studies (Tardif et al., 1993, 1995, 1997; Loizou et al., 1999; Jonsson and Johanson, 2001a, 2002; Jonsson et al., 2001; Krishnan et al., 2002; McNally et al., 2012). Data generated in controlled human studies are not only useful for investigating the structure and quantitative outputs of PBPK models but also the biological plausibility and variability of parameters for which measured values are not available (Jonsson and Johanson, 2001a,b, 2002; Jonsson et al., 2001).

In this report we present the BM results from a controlled human study where volunteers were exposed to a binary mixture of ethanol and *m*-xylene. This combination was selected because *m*-xylene metabolism is known to be inhibited by ethanol during and post-acute ingestion (Riihimäki et al., 1982; Wilson et al., 1983; MacDonald et al., 2002). We also describe PBPK models for exposure to *m*-xylene alone and the binary mixture of ethanol and *m*-xylene. A global sensitivity analysis (SA) of both PBPK models based on the workflow proposed by McNally et al. (2011) is conducted, and the results from the two models are compared and contrasted. Finally we present the results from model calibration whereby the sensitive uncertain parameters in the PBPK model for the exposure to a binary mixture of ethanol and *m*-xylene were tuned using a Bayesian approach (McNally et al., 2012) such that model predictions and observed BM data were consistent.

## Materials and Methods

### Volunteers

The UK Health and Safety Executive Research Ethics Committee approved the study. Volunteers, who all were Health and Safety Laboratory staff, provided written informed consent before participating. Eight volunteers, seven male and one female (aged 29–54; **Table 1**) took part and were in good health at the time of the study, did not suffer from respiratory disease, and were not on any medications. Medical assessments were made immediately before the start and at the end of each experiment, to ensure that each volunteer was fit to participate and then to be discharged, respectively. The medical supervisor was present throughout the exposure period. All volunteers were asked to refrain from alcohol consumption for at least 72 h before entering the study. Body mass, height, body mass index (BMI), mass of body fat, resting minute volume, mean urine flow, urinary creatinine concentration and *m*-xylene blood:air partition coefficient (PC) for each volunteer was measured (**Table 1**). Body fat was measured using a bio-electrical impedance analyser (Bodystat 1500 Ltd., Isle of Man, UK) and by skinfold thickness measurements (Holtain callipers, Holtain Ltd., Crymych, UK). The value for mass of body fat used was the mean of the two techniques. Resting minute volume was measured using a Morgan Medical Pulmolab TF 501 apparatus at the Respiratory Function Unit of the Royal Hallamshire Hospital, Sheffield. The alveolar ventilation rate was assumed to be 70% of minute volume (Brown et al., 1997).

**Table 1:** Measured parameters.

Volunteer	Age	Body weight (kg) (BW)	Height (m)	BMI (kg/m^2^)	Mass of body fat (% BW) (VFAC)	Resting alveolar ventilation rate (l/h) (QPMC)	Average urine flow (l/h) (R_urine_)	Average urinary creatinine (mmol/l) (CREmmol)	*m*-xylene Blood:air partition coefficient (PBAXYL)
A	54	79	1.68	28.00	0.218	383.3	0.070	14.8	15.1
B	51	61.5	1.78	19.32	0.192	409.3	0.125	7.30	16.8
C	47	89	1.91	24.40	0.169	477.5	0.090	14.9	11.4
D	48	85	1.75	27.80	0.263	362.7	0.091	13.0	18.0
E	29	76	1.85	22.20	0.130	327.2	0.088	12.5	26.5
F	25	76	1.83	22.70	0.162	352.6	0.055	14.8	20.2
G	41	75	1.70	26.00	0.179	462.2	0.076	12.8	–
H	29	68	1.70	23.50	0.299	348.6	0.074	10.2	21.6
Mean		76.2	1.78	24.24	0.202	390.4	0.083	12.5	18.5
SD		8.73	0.08	2.95	0.056	54.89	0.021	2.70	4.90
CV		0.115	0.05	0.122	0.277	0.141	0.247	0.212	0.26

### Chemicals

Absolute ethanol, *m*-xylene (99%) and 3-methylhippuric acid (98%) were obtained from Aldrich Chemical Co. (Dorset, UK). All other chemicals used were reagent grade or higher.

### Exposure Protocol

Exposures were performed in the Health and Safety Laboratory Controlled Atmosphere Facility (CAF), a purpose built room 8 m^3^ in volume. Atmospheres of *m*-xylene vapor were generated by purging *m*-xylene-filled bubblers with compressed air into the CAF. The atmospheric concentration within the CAF was monitored continuously by a Miran infra-red spectrophotometer (calibrated by an internal, closed-loop system) and by gas chromatography (Varian 6000, with a 0.05 m % 0.5 mm i.d., with 5% OV10, 100–120 mesh Chrom CHP packing; injector temperature 120°C; N_2_ carrier gas flow rate 40 mL min^-1^; oven temperature 60°C) with flame ionization detection (detector temperature 200°C, H_2_ flow rate 25 mL min^-1^, air flow rate 300 mL min^-1^, calibration using a gas sampling valve against a standard atmosphere). The CAF temperature was maintained at 25°C and 30% humidity for all experiments.

Groups of four volunteers were exposed for 4 h on two separate occasions to a target concentration of 50 ppm *m*-xylene vapor. The first exposure was to *m*-xylene only. The second exposure, which was scheduled 1 month after the first, was started 30 min after the volunteers had ingested 0.8 g kg**^-^**^1^ ethanol, diluted in fruit juice. The actual measured exposure concentrations for the duration of the experiments were: 45.5 ± 5.7 and 44.1 ± 6.9 ppm, for the exposures without prior ethanol ingestion, and 45.1 ± 4.7 and 43 ± 10.3 ppm, with prior ethanol ingestion, respectively. The exposure protocols and BM are summarized in **Table 2**. The dose of ethanol required to inhibit CYP2E1 was obtained from a previous study, which reported blood *m*-xylene concentrations in human volunteers, with and without, prior ingestion of 0.8 g kg^-1^ ethanol (Riihimäki et al., 1982). The data could be interpreted as having demonstrated inhibition of *m*-xylene metabolism (Riihimäki et al., 1982). However, inhibition of CYP2E1 by 0.8 g kg^-1^ ethanol was confirmed in this study by measuring the hydroxylation of the probe substrate, chlorzoxazone (Loizou and Cocker, 2001). The individual amounts of ethanol and final volume of fluid ingested are listed in **Table 3**.

**Table 2:** Exposure protocols and biological monitoring.

	Exposure concentration (ppm)		Biological monitoring					
			Blood *m*-xylene		Breath *m*-xylene		Urinary MHA (g/g creatinine)	
Volunteer	Ethanol pre-treatment							
	No	Yes	No	Yes	No	Yes	No	Yes
A	45.5	43.0	√	√	√	√	√	√
B	45.5	43.0	×	√	√	√	√	√
C	45.5	45.1	√	√	√	√	√	√
D	45.5	43.0	√	√	√	√	√	√
E	44.1	45.1	×	√	√	√	√	√
F	44.1	45.1	×	√	√	√	√	√
G	44.1	43.0	×	√	√	√	√	√
H	44.1	45.1	×	√	√	√	√	√

**Table 3:** Individual ethanol doses and calculated gastric emptying rates.

Volunteer	Weight (kg)	Amount of ethanol (g) for dose of 0.8 g/kg	Volume of absolute ethanol (ml)	Final volume diluted 1:4 with fruit juice (ml)	Half-life of gastric emptying (h)	Gastric emptying rate *K_e(max)_* (h^-1^)
A	79	63	80	320	0.122	5.67
B	61.5	49	62	248	0.093	7.49
C	89	71	90	360	0.139	4.99
D	85	68	86	344	0.132	5.24
E	76	61	77	308	0.117	5.91
F	76	61	77	308	0.117	5.91
G	75	60	76	304	0.116	5.99
H	68	54	69	276	0.104	6.67
Mean	76.2	60.9	77.1	308.5	0.117	5.99
SD	8.73	7.04	8.85	35.4	0.015	0.791
CV	0.115	0.116	0.115	0.115	0.128	0.132

Venous blood samples were taken at 0, 0.3, 0.7, 1, 2, 3, 4, 4.33, 4.67, 5, 6, 7, 8, and 23 h. The blood data were separated into two sets corresponding to measurements made on different days. Unfortunately, five from eight samples from the exposures without prior administration of ethanol were deemed unreliable due to the possible loss of volatilised *m*-xylene because of imperfect sealing of sample vials (McNally et al., 2012). Therefore, it was only possible to compare venous blood concentrations of *m*-xylene, with and without prior administration of ethanol, in just three volunteers.

Exhaled air samples were taken at 0, 4.017, 4.33, 4.67, 5, 6, 7, 8, and 24 h. Urine samples were taken at 0, 4, 6, 8, 10, 12, 14, 24, 27, 31 h.

### Biological Monitoring

Blood (CV_xyl_) and exhaled alveolar *m*-xylene (CX_ppm_) and urinary 3-methylhippuric acid (MHA) concentrations (C_urine_) were analyzed. Volunteers provided blood samples from the antecubital vein via an indwelling soft cannula. Samples were taken prior to entering the exposure facility and at hourly intervals during exposure, then every 20 min for the first hour after exiting the exposure facility before returning to hourly intervals for the next 3 h. A single sample was collected the next day at 23 h after the start of the study. Blood samples were stored in sealed vials containing EDTA as anticoagulant (48 h, maximum) at 4°C as whole blood until analyzed. Blood and urine samples were analyzed in duplicate as follows: a 250 μL sample was added to 750 μL of H_2_O in a 10 mL headspace vial, which was capped with a PTFE-lined rubber septum. The sample was then incubated and continually stirred at 65°C for 10 min. A 1 mL headspace aliquot was taken using a pre-warmed (75°C) gas-tight syringe (Fisons HS800 headspace sampler) and analyzed by gas chromatography (Carlo Erba GC8000; column BP-5, 25 m 0.32 mm i.d., 5 μm film) and mass spectrometry (Fisons MD800 MS) operating in selected ion monitoring mode using positive electron ionization (m/z M^+^ 106). The limit of detection of the assay was 0.1 μmol L^-1^ with intra- and inter-assay coefficients of variation (CVs) of 5 and 10%, respectively.

End tidal breath samples (alveolar air) were taken and analyzed according to the method of (Dyne et al., 1997) and as described previously (Loizou et al., 1999).

Urine volume was recorded and samples stored at -20°C until analyzed. The major metabolite, 3-methylhippuric acid (3-MHA) was measured in the urine to assess the rate of biotransformation and elimination of *m*-xylene. A 0.5 mL sample of urine was mixed with 0.5 mL methanol and analyzed by HPLC (Hewlett Packard 1050 Series, with auto-sampler, pump, degasser; column 3 μm ODS, 100 4.6 mm) with a mobile phase of 0.1% acetic acid:methanol (85:15 with gradient elution), using diode array detection at a detection wavelength of 230 nm. The limit of detection of the assay was 40 μmol L^-1^, with intra- and inter-assay CVs of 2 and 5%, respectively.

### PBPK Models

Two PBPK models, for *m*-xylene and ethanol, were interconnected at the level of the liver as described by Krishnan et al. (2002), where the chemicals compete for metabolism by the same enzyme. Only liver metabolism was described since hepatic microsomal enzyme activities are generally significantly higher than those in other organs (Pacifici et al., 1988). Both models were described previously (Loizou and Spendiff, 2004; McNally et al., 2012). The ethanol model incorporated a description of the retardation of gastric emptying due to a concentration-dependent inhibition of gastric peristalsis by ethanol (Loizou and Spendiff, 2004). The *m*-xylene model, included a bladder compartment to simulate fluctuations in metabolite concentration in the urine (McNally et al., 2012), was modified to describe competitive, non-competitive and un-competitive inhibition of *m*-xylene metabolism by ethanol. Simulations of venous blood concentrations of *m*-xylene were run using each of the inhibition mechanisms. Competitive inhibition was considered to provide the best fit to the data by simple visual inspection. Initial, deterministic simulations were conducted using the parameters listed in **Tables 3–6**.

**Table 4:** Anatomical, physiological and kinetic constants and parameters common to both models.

Parameter	Abbreviation	Value	Distribution
Molecular mass *m*-xylene (g/mol)	MW_xyl_	106.17	–
Molecular mass MHA (g/mol)	MW_MHA_	193.2	–
Body mass (kg)	BW		Normal BW∼N(76.2,(8.73)^2^)
Vascularised tissue (proportion of body mass)	VT	0.91	–
Cardiac output (L h^-1^ BW^-0.75^)	QCMC	13.8	Normal QCMC∼N(13.8,(2.5)^2^)
Microsomal protein yield per gram wet weight liver (mg g^-1^)	MPY	34	Lognormal ln(MPY)∼N(37,(2.9)^2^)
**Gas exchange**		
Respiratory rate (L h^-1^)	QPMC	390.4	Normal QPMC∼N(390.4,(54.9)^2^)
Respiratory dead space (proportion respiratory rate)	DS	0.3	–
**Tissue blood flow as a fraction of cardiac output**		
Rapidly perfused	QRPDC	0.48	–
Slowly perfused	QSPDC	0.22	Uniform QspdC ∼U(0.2–0.35)
Adipose	QFAC	0.05	Normal QfaC∼N(0.053,(0.003)^2^)
Liver	QLIC	0.25	Normal QliC∼N(0.271,(0.01)^2^)
**Tissue mass as a fraction of body mass**		
Rapidly perfused	VRPDC	0.09	–
Slowly perfused	VSPDC	0.604	–
Adipose	VFAC	0.19	Lognormal ln(VfaC)∼N(-1.59,(-2.88)^2^)
Liver	VLIC	0.0257	Normal VliC∼N(0.036,(0.01)^2^)
**Bladder compartment**		
Rate of urine production (L h^-1^)	R_urine_	0.07	Normal R_urine_∼N(0.083,(0.021)^2^)
Urinary creatinine concentration (mmol L^-1^)	CREmmol	12.5	Normal CREmmol∼N(12.5,(2.7)^2^)
First-order elimination rate constant (h^-1^)	K_1_	20	Uniform K_1_∼U(5–20)

**Table 5:** Parameters specific to the *m*-xylene model.

Molecular masses		
Molecular mass *m*-xylene (g/mol)	MW_xyl_	106.17	–
Molecular mass MHA (g/mol)	MW_MHA_	193.2	–
**Metabolism (liver)**			
*In vitro* Michaelis constant (mMol L^-1^)	K_M2E1xyl_	11.8	Normal K_M_ ∼N(11.8,(1.4)^2^)
*In vitro* maximum rate of metabolism (pmol min^-1^ mg^-1^ microsomal protein)	V_max2E1xyl_	895	Normal V_max_ ∼N(895,(68)^2^)
Inhibitory rate constant (mg L^-1^)	KI	10	Uniform KI∼U(1–20)
**Partition coefficients**			
Blood:air partition coefficient	PBAXYL	18.5	Normal Pba∼N(18.5,(4.9)^2^)
Rapidly perfused	PRPDAXYL	117	Uniform Prpda∼U(50–150)
Slowly perfused	PSPDAXYL	53	Uniform Pspda∼U(40–80)
Adipose	PFAAXYL	1874	Uniform Pfaa∼U(1400–2200)
Liver	PLIAXYL	279	Uniform Plia∼U(150–350)

**Table 6:** Parameters specific to the ethanol model.

Parameter	Abbreviation	Value	Distribution
Molecular mass ethanol (g/mol)	MW_eth_	46.07	–
**Gastric compartment**		
Oral dose (mg)	PORALDOSE	800	Uniform PORALDOSE ∼U(480–720)
Drink time (h)	DRINKTIME	0.25	Uniform DRINKTIME ∼U(0.2–0.3)
Drink volume (L)	DRINKVOL	0.5	Uniform DRINKVOL ∼U(0.4–0.6)
Stomach permeability (h^-1^)	BELLYPERM	0.685	Uniform BELLYPERM ∼U(0.548–0.822)
Gut permeability (h^-1^)	GIPERM	21.1	Uniform GIPERM ∼U(20.8–30.12)
Maximum emptying rate (h^-1^)	KE_max_	10.2	Uniform KE_max_ ∼U(8.16–12.24)
Minimum emptying rate (h^-1^)	KE_min_	0.005	Uniform KE_min_ ∼U(0.004–0.006)
**Tissue mass as a fraction of body mass**		
Stomach	VSTC	0.02	Uniform VstC ∼U(0.016–0.024)
Gut	VGUC	0.085	Uniform VguC ∼U(0.068–0.102)
**Tissue blood flow as a fraction of cardiac output**		
Stomach	QSTC	0.01	Uniform QstC ∼U(0.0056–0.0084)
Gut	QGUC	0.17	Uniform QguC ∼U(0.136–0.204)
**Metabolism (liver)**		
*In vitro* Michaelis constant (mMol L^-1^)	K_M2E1eth_	12.5	Uniform K_M2E1_∼U(9.98–14.9)
*In vitro* maximum rate of metabolism (pmol min^-1^ mg^-1^ microsomal protein)	V_max2E1eth_	12060	Uniform V_max2E1_∼U(9649–14473)
*In vitro* Michaelis constant (mMol L^-1^)	K_Mαα_	4.2	Uniform K_Mαα_∼U(3.36–5.04)
*In vitro* maximum rate of metabolism (pmol min^-1^ mg^-1^ microsomal protein)	V_maxαα_	18327	Uniform V_maxαα_ ∼U(14661–21993)
*In vitro* Michaelis constant (mMol L^-1^)	K_Mββ_	0.05	Uniform K_Mββ_ ∼U(0.04–0.06)
*In vitro* maximum rate of metabolism (pmol min^-1^ mg^-1^ microsomal protein)	V_maxββ_	5934	Uniform V_maxββ_ ∼U(4747–7121)
*In vitro* Michaelis constant (mMol L^-1^)	K_Mγγ_	0.63	Uniform K_Mγγ_ ∼U(0.5–0.54)
*In vitro* maximum rate of metabolism (pmol min^-1^ mg^-1^ microsomal protein)	V_maxγγ_	11895	Uniform V_maxγγ_ ∼U(9516–14274)
**Partition coefficient**		
Blood:air partition coefficient	Pba_eth_	1265	Uniform Pba_eth_ ∼U(1012–1518)
Rapidly perfused	Prpda_eth_	0.95	Uniform Prpda_eth_ ∼U(0.76–1.14)
Slowly perfused	Pspda_eth_	0.80	Uniform Pspda_eth_ ∼U(0.64–0.96)
Adipose	Pfaa_eth_	0.11	Uniform Pfaa_eth_ ∼U(0.088–0.132)
Liver	Plia_eth_	0.81	Uniform Plia_eth_ ∼U(0.648–0.972)
Stomach	Pst_eth_	0.81	Uniform Pst_eth_ ∼U(0.648–0.972)
Gut	PGU_eth_	0.81	Uniform PGU_eth_ ∼U(0.648–0.972)

### Partition Coefficients

Pre-exposure blood samples were taken from each volunteer for the determination of the blood:air *m*-xylene PCs. The tissue:air partition coefficients were determined as described previously (Loizou et al., 1999) according to the method of Gargas et al. (1989). Tissue:blood PCs were determined by dividing the tissue:air PCs by the blood:air PC. Surrogate PCs for the slowly and rapidly perfused compartments were obtained from the literature (Lapare et al., 1993).

Rat tissue:blood PCs for ethanol determined by Kaneko et al. (1994) were used as surrogates for human tissue and the human blood:air PC was obtained from (Pastino et al., 1997) as described previously (Loizou and Spendiff, 2004).

### Parameter Distributions

Anatomical and physiological parameter distributions used for global SA and Markov-chain Monte Carlo (MCMC) simulations were obtained from the freely available web-based application PopGen, which is a virtual (healthy) human population generator (^1^McNally et al., 2012). A human population, comprising 50% male and 50% female, white Caucasians, age range 16–65, height range 140–200 cm, body mass indices 18.5–30 was generated to encompass the characteristics of the volunteers that took part in the study. Most anatomical and physiological parameters were common to both the *m*-xylene and ethanol models (**Table 4**). The following parameters differed; metabolic rate constants, PCs and those describing the inhibition of gastric motility (**Tables 5 and 6**).

Apart from the blood:air PC for *m*-xylene, no distributions were available for the PCs, therefore, uniform distributions were assigned and the ranges set were considered reasonable assumptions. *VspdC* and *VrpdC*, the masses of the slowly and rapidly perfused tissues respectively, were not included in the SA because they are aggregated compartments from which organs and tissues are subtracted when discretely defined to maintain tissue mass balance. Uniform distributions were assigned to the ethanol-model-specific parameters as only point values were originally reported (Pastino et al., 2000; Loizou and Spendiff, 2004). The model was re-parameterised as proposed by Gelman et al. (1996) to ensure that mass balance and blood blow constraints were not violated.

### Sensitivity Analysis

A two-phased global SA was conducted following the workflow proposed in McNally et al. (2011). In the first phase the Morris Screening test was used to eliminate the parameters with a negligible effect on model output. The Morris test results are not discussed in detail; however, this reduced the number of varying parameters in the binary model from 47 to 10 or 12. These less sensitive parameters were subsequently fixed at central values. In the second phase a reduced number of parameters were analyzed using the extended Fourier Amplitude Sensitivity Test (eFAST) to obtain quantitative variance-based measures of sensitivity. eFAST produces two sensitivity indices for each parameter: the main effect sensitivity index *S_i_* quantifies the importance of the parameter in isolation whereas the total effect sensitivity index *S_Ti_* additionally quantifies the interactions with other model parameters. The difference between *S_Ti_* and *S_i_* is therefore a measure of interactions alone. The sensitivity indices were calculated over a specified portion of the concentration-time simulation, these were: 0–8 h for CV_xyl_, 0–10 h for CX_ppm_, and 0–15 h for C_urine_. In each case, the specified portion of the concentration-time simulation was selected to investigate the parameter sensitivities during the phases of greatest change, i.e., uptake, distribution and elimination and to avoid the regions where sensitivity measures reflect sampling variability and noise rather than important model structure (McNally et al., 2011). SA results were computed on a fine timescale. The sensitivities of CV_xyl_ and CX_PPM_ at the 3 and 6-h time points within the distribution and elimination phases respectively, and at 6 and 10 h for C_urine_ in the early and latter urinary elimination phases are reported as broadly representative of the SA results: from 0 to 4 h ‘absorption into the body’ and the period after 4 h ‘elimination from the body’ for CV_xyl_ and CX_PPM_, and 4–8 h ‘rapid elimination’ and after 8 h ‘return to baseline’ for C_urine_. Lowry plots (McNally et al., 2011, 2012) are used to visualize results from the SA at these time points.

### Calibration

Calibration describes the process of tuning the PBPK model parameters such that the time-varying predictions of model output CV_xyl,_ CX_PPM_, and C_urine_ are consistent with observed (in this case BM) data. The most sensitive parameters were identified by SA therefore the computational burden of calibration was reduced by focussing on just these sensitive parameters. The measured parameters, body mass (BW), fat mass (VfaC), resting alveolar ventilation rate (QPMC), urine flow (Rurine), and urinary creatinine (CREmmol) were known for each individual (**Table 1**) and therefore fixed. Although the *m*-xylene blood:air PC (Pbaxyl) was measured this parameter was also calibrated. This is because blood:air PCs are known to vary with consumption of food (Jonsson et al., 2001) and could therefore change significantly from within day and day to day. The “*in vivo*” K_M_ value, 12.5 mM (575 mg L^-1^) for the hepatic microsomal oxidation of ethanol was used as an initial value for the inhibitory rate constant KI, for the inhibition of hepatic cyp450 *m*-xylene metabolism (Derr, 1993). However, this value was ineffective, therefore, KI was also calibrated.

The most sensitive unknown parameters as identified and quantified in SA were calibrated using a Bayesian approach described previously (McNally et al., 2012). Briefly, the differences between model predictions and BM data were assumed to follow a lognormal distribution. A MCMC algorithm was used to sample values for the most sensitive unknown parameters. The posterior modal parameter set that is, the parameter set which results in the lowest discrepancy between predictions and BM data was extracted from the MCMC output and compared with the BM data so the overall adequacy of the model could be assessed.

### Software

The numerical solutions to the model equations were obtained using acslX Libero version 3.0.1.6 (AEgis Technologies^2^). The M functions for eFAST and MCMC modeling included with the acslX Optimum suite of tools were adapted for use in this study. Lowry plots were created using a bespoke web application developed by the authors^3^. All other figures were created using GraphPad Prism 4 for Windows^4^ A Dell Optiplex 755 Intel Core(TM)2 Duo CPU 3.00GHz 2.00GB RAM was used for all simulations in this study.

## Results

### Sensitivity Analysis

**FIGURE 1:**
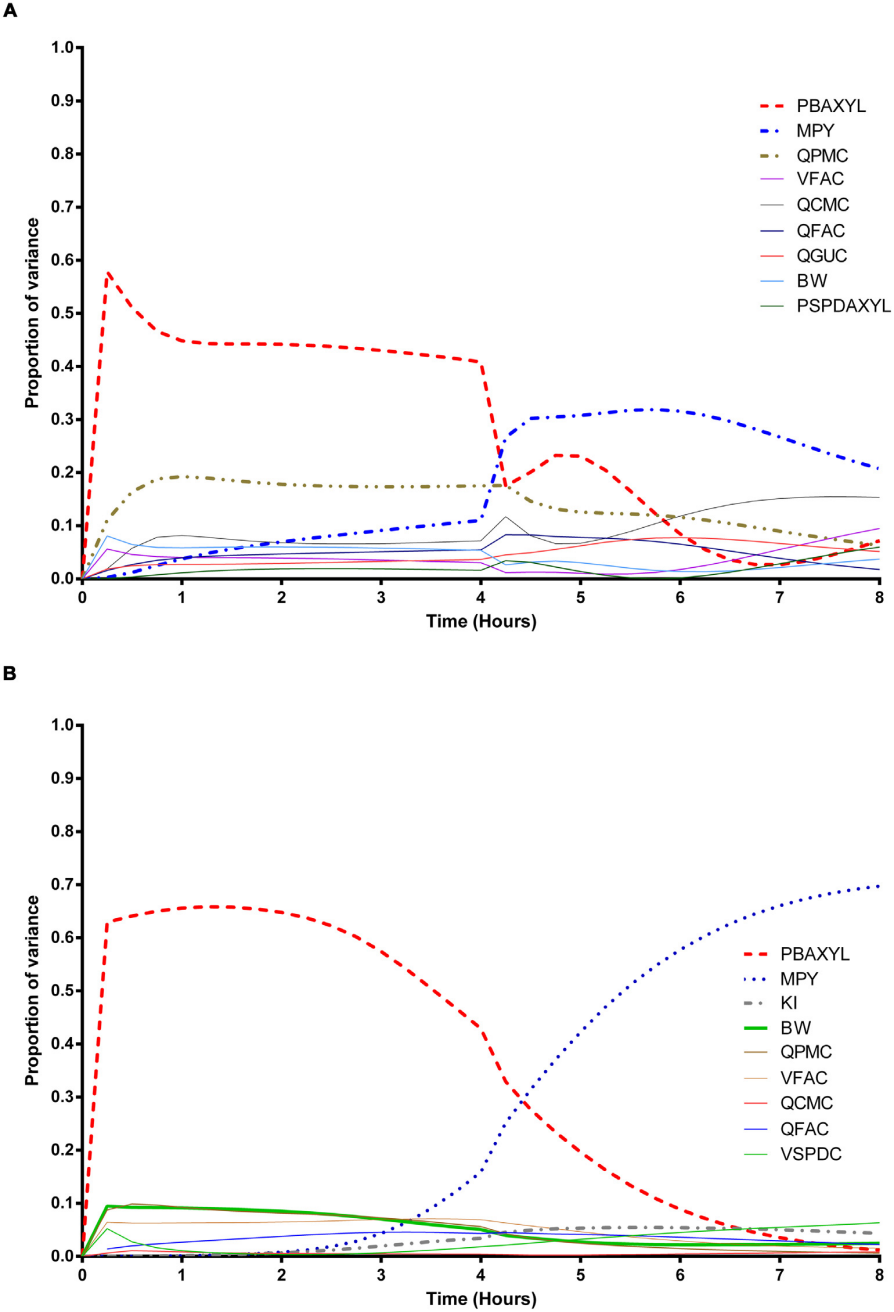
**Parameter main effects.** Change in proportion of variance over time for venous blood concentration of *m*-xylene (CV_xyl_): **(A)** without inhibition of *m*-xylene metabolism and **(B)** with inhibition of *m*-xylene metabolism. The broken lines represent the most dominant parameters over the entire simulation. Parameter abbreviations are listed in **Table 4**.

**FIGURE 2:**
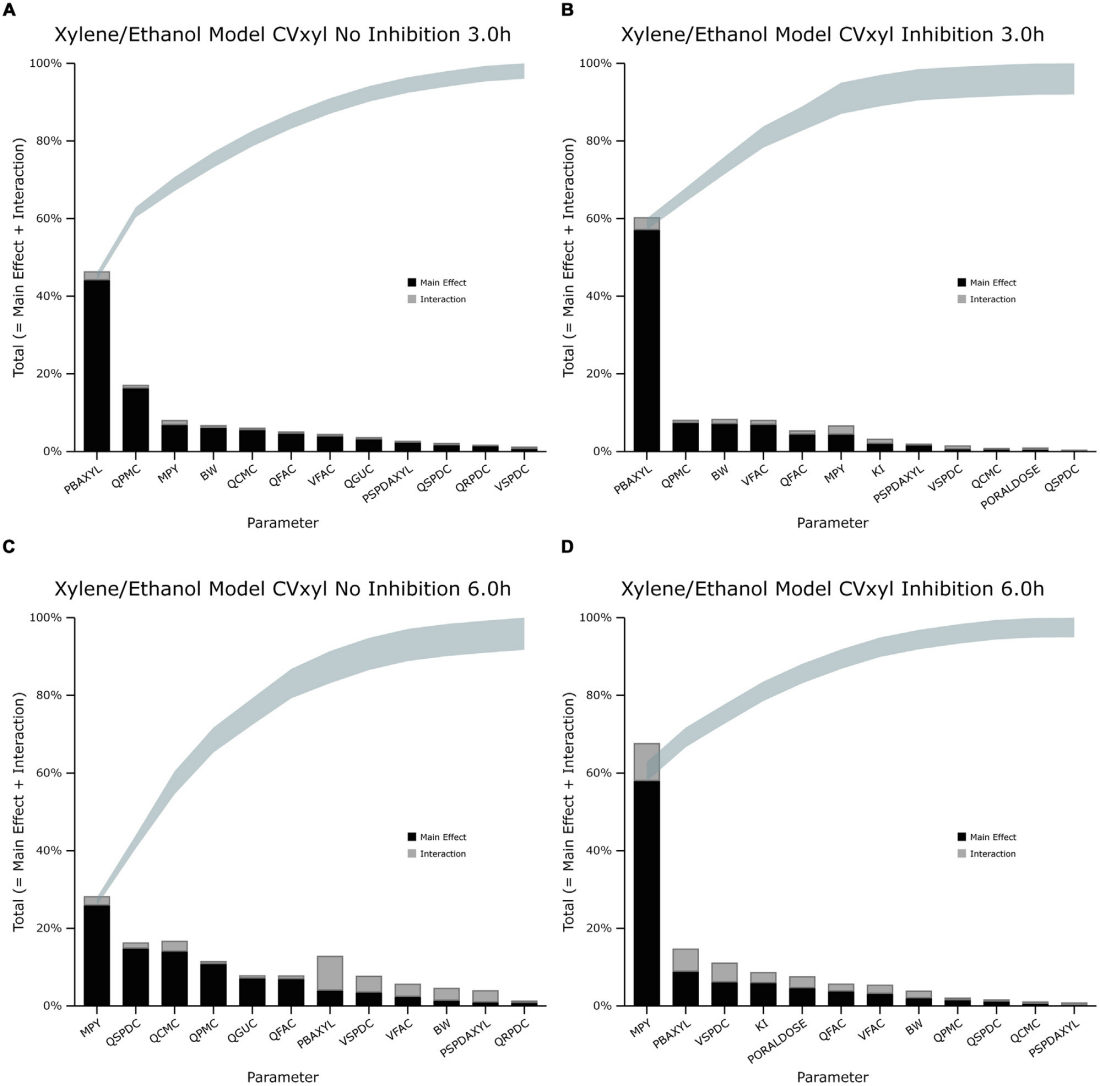
**Lowry plot of the eFAST quantitative measure for variance in venous blood *m*-xylene concentration (CV_xyl_). (A,C)** Shows the total effects without inhibition of *m*-xylene metabolism and **(B,D)**, with inhibition at 3 and 6 h after initiation of the simulation. Parameter abbreviations are listed in **Table 4**.

**FIGURE 3:**
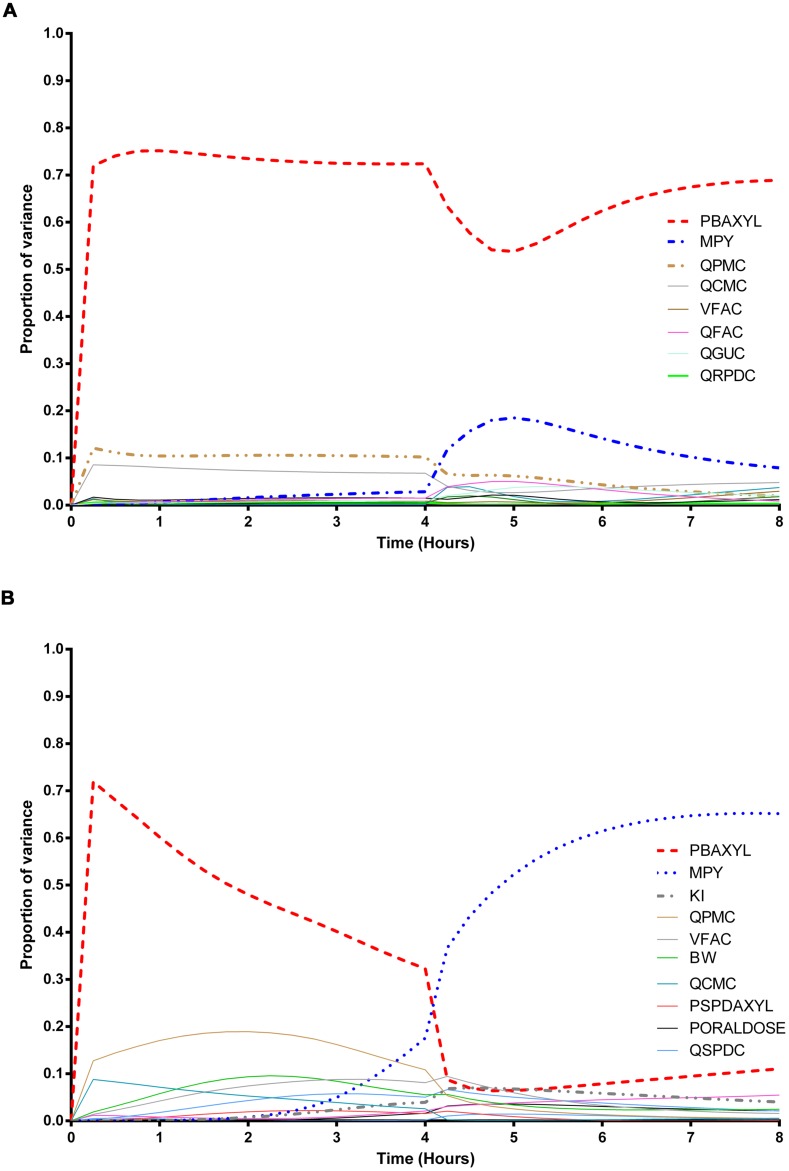
**Parameter main effects.** Change in proportion of variance over time for end-exhaled concentration of *m*-xylene (CX_ppm_): **(A)** without inhibition of *m*-xylene metabolism and **(B)** with inhibition of *m*-xylene metabolism. The broken lines represent the most dominant parameters over the entire simulation. Parameter abbreviations are listed in **Table 4**.

**FIGURE 4:**
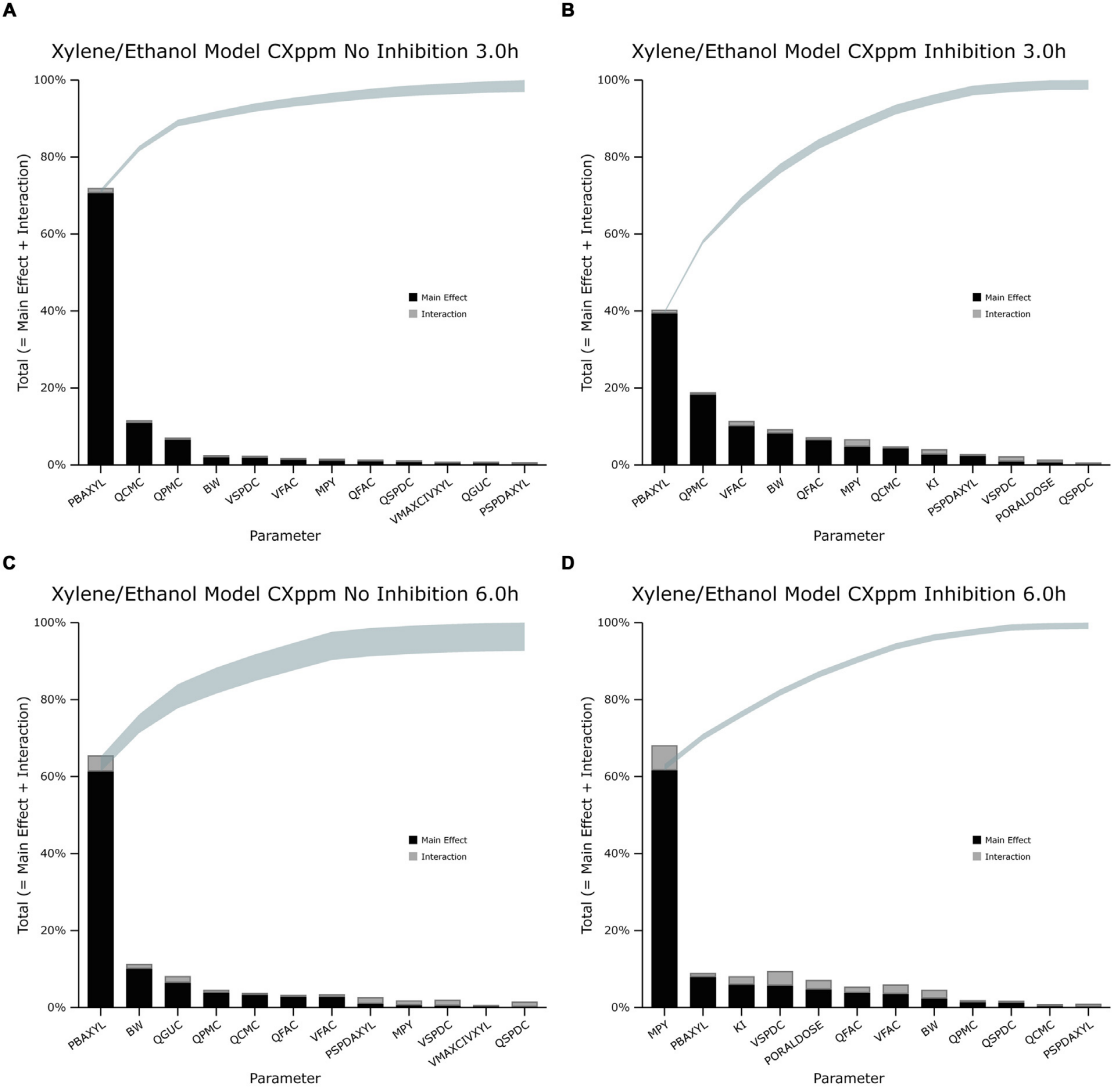
**Lowry plot of the eFAST quantitative measure for variance in venous blood *m*-xylene concentration (CX_ppm_). (A,C)** Shows the total effects without inhibition of *m*-xylene metabolism and **(B,D)**, with inhibition at 3 and 6 h after initiation of the simulation. Parameter abbreviations are listed in **Table 4**.

**FIGURE 5:**
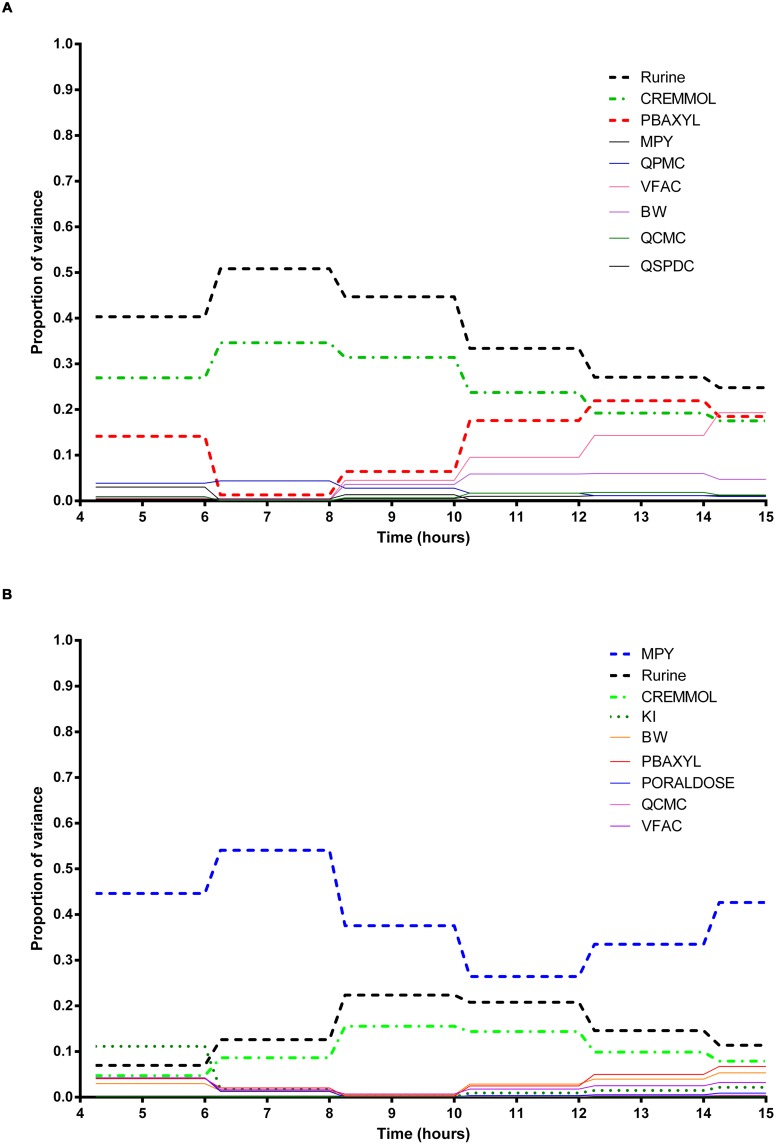
**Parameter main effects.** Change in proportion of variance over time for urinary excretion of 3-methylhippuric acid (C_urine_): **(A)** without inhibition of *m*-xylene metabolism and **(B)** with inhibition of *m*-xylene metabolism. The broken lines represent the most dominant parameters over the entire simulation. Parameter abbreviations are listed in **Table 4**.

**FIGURE 6:**
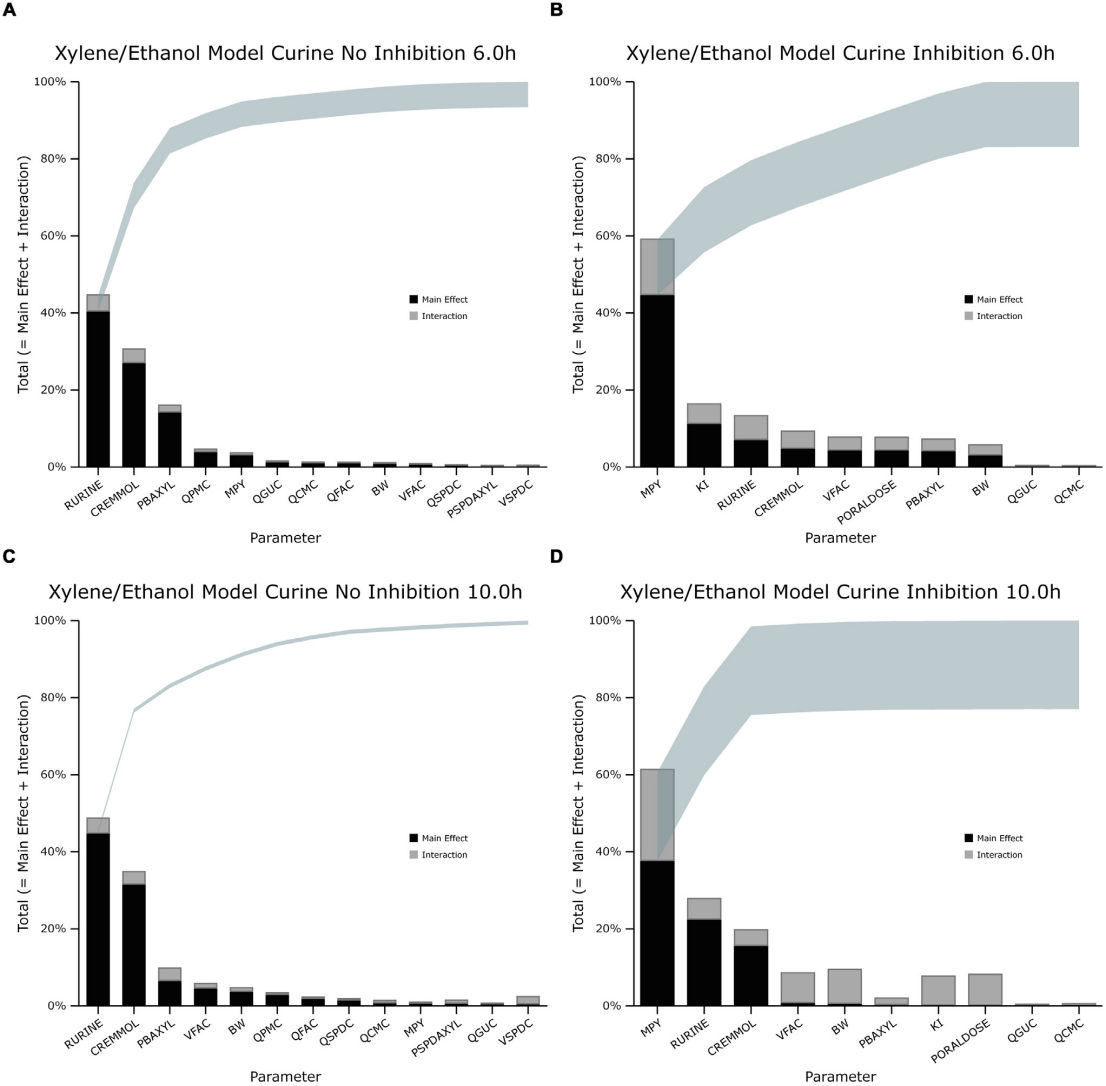
**Lowry plot of the eFAST quantitative measure for variance in venous blood *m*-xylene concentration (Curine). (A,C)** Shows the total effects without inhibition of *m*-xylene metabolism and **(B,D)**, with inhibition at 6 and 10 h after initiation of the simulation. Parameter abbreviations are listed in **Table 4**.

The information obtained from global SA is presented using two figures for each of the studied model outputs. **Figures 1, 3 and 5** show the change in proportion of variance of the main effects *S_i_*, over time for the parameters selected for eFAST for CV_xyl_, CX_ppm_, and C_urine,_ respectively. In each figure Panel A shows the main effects of parameters with no inhibition, and Panel B with inhibition by ethanol. The most dominant parameters are represented by broken lines. The inhibitory rate constant KI, was also represented as a broken line irrespective of contribution to variance. **Figures 2, 4 and 6** show plots of main effects and interactions terms at 3 and 6 h for CV_xyl_, CX_ppm_ and at 6 and 10 h for C_urine_, respectively: the chosen time points are snapshots of the sensitivities in the distribution and elimination phases, respectively. In each figure Panel A shows the main effects of parameters with no inhibition, and Panel B with inhibition by ethanol.

### Venous Blood *m*-Xylene

In the absence of inhibition of *m*-xylene metabolism, PBAXYL was initially dominant in simulations of CV_xyl_ (**Figure 1A**) with a gradual decrease to the end of the exposure period at 4 h. The main effect for QPMC was also large and near constant after the first hour of exposure. The main effect for MPY steadily increased in importance throughout the simulated 4 h. The main effects of the three most influential parameters from 0.5 to 4 h accounted for 68.9 to 72.3%^5^ of variance with PBAXYL, the most dominant, ranging from 40.8 to 44.8% (**Figure 1A**). The Lowry plot shown in **Figure 2A** indicates that interactions were generally small. The main effects of the three most influential parameters from 5 to 8 h accounted for 48.3–66.4% of variance with MPY, the most dominant, ranging from 20.7 to 31.6% (**Figure 1A**). In summary, PBAXYL and MPY were the most dominant parameters from 1.0 to 8 h together accounting for 27.9–53.9% of variance. No other individual parameter accounted for greater than 19.2% of variance at any time.

In the presence of inhibition of *m*-xylene metabolism the main effects of the three most influential parameters from 0.5 to 4 h accounted for 65.7–83.9% of variance (**Figure 1B**). The main effect of PBAXYL was the dominant parameter between 0.5 and 4 h, accounting for between 42.9 and 65.6% of variance. The main effect of PBAXYL declined sharply in the last hour of the exposure. CV_xyl_ had reduced sensitivity to both QPMC and MPY compared to the simulations without inhibition (**Figures 1A,B**) for the duration of the exposure (0–4 h). **Figure 1B** shows that the main effect of MPY was close to zero in the first 2 h and began to sharply increase in importance after 3 h of exposure. The interactions between inputs were still modest between 0.5 and 4 h however, **Figure 2B** indicates some small interactions between the parameters governing the rate of metabolism of *m*-xylene in this time period (particularly MPY and KI). Whilst the detailed analysis at 3 h is intended to be broadly representative of overall parameter sensitivity in the first 4 h, it is clear from **Figure 1B** that results after 3 h are time sensitive, due to rapid changes in the sensitivity to PBAXYL and MPY and interaction terms. The main effects of the three most influential parameters from 5 to 8 h accounted for 67.2–80.4% of variance with MPY, the most dominant, ranging from 42.2 to 69.7% (**Figure 1B**). The interaction terms were more significant in this period as indicated by the width of the plume (**Figure 2D**). Two clusters of interacting parameters were observed: the first concerned interactions between PBAXYL, BW and VSPDC and VFAC (as seen in both **Figures 2C,D**); the second concerning interactions between parameters governing metabolism (MPY, PORALDOSE, and KI).

The switch from PBAXYL to MPY from exposure to post-exposure periods is consistent with a decrease in the hepatic concentration of ethanol which, combined with the relatively high K_M_ and therefore, lower affinity for ethanol metabolism by the cytochrome P450 (CYP450) enzyme compared to *m*-xylene, is consistent with decreased competition with *m*-xylene for metabolism. MPY can be seen as an indication of the contribution of metabolism to variance because MPY significantly affects the magnitude of V*_max_* in the *in vitro*–*in vivo* scaling calculation.

### Exhaled *m*-Xylene

In the absence of inhibition of *m*-xylene metabolism PBAXYL was the dominant parameter throughout the simulation period with the main effect accounting for between 53.7 and 75.2% of variance (**Figure 3A**). QPMC was also an important parameter throughout the simulation period whereas MPY increased in importance after the exposure terminated (at 4 h). The SA identified the same subset of important parameters for CX_ppm_ as for CV_xyl_ although the contributions to variance differed. The detailed analysis at 3 h (**Figure 4A**) showed interactions were negligible. The detailed analysis at 6 h showed the SA of CX_ppm_ broadly followed the results from CV_xyl_ with the same influential parameters observed and interactions between PBAXYL, BW and the VSPDC and VFAC tissues (**Figure 4C**).

In the presence of inhibition of *m*-xylene metabolism the SA showed that whilst PBAXYL remained the most important parameter in the exposure period the main effect accounted for a smaller proportion of variance ranging from 32.3 to 72.0% (**Figure 3B**). The main effects of the three most influential parameters from 1.0 to 4 h accounted for 60.7–84.4% of variance. **Figure 3B** shows that the main effect of MPY was close to zero in the first 2 h and began to sharply increase in importance after 3 h of exposure. The detailed analysis in **Figure 4B** showed some small interactions, principally between parameters related to metabolism (MPY, PORALDOSE, and KI).

The main effects of the three most influential parameters from 5 to 8 h accounted for 65.5–81.7% of variance with MPY, the most dominant, ranging from 52.3 to 65.1% (**Figure 3B**). The interactions between parameters governing metabolism were larger in this period (**Figure 4D**).

In summary, PBAXYL and MPY were the most dominant parameters from 1.0 to 8 h together accounting for 45.3–76.2% of variance. No other individual parameter accounted for greater than 18.9% of variance at any time.

### Urinary 3-Methylhippuric Acid

In the absence of inhibition of *m*-xylene metabolism, R_urine_ and CREmmol were the most dominant parameters governing variance in urinary excretion of 3-MHA up to 8 h post-exposure when PBAXYL began to exceed CREmmol, albeit slightly (**Figure 5A**) and BW and VFAC increased in importance^6^. The main effects of the two most influential parameters from 4 to 12 h, R_urine_ and CREmmol accounted for 57.1–85.4% of variance (**Figure 5A**). No other individual parameter accounted for greater than 17.6% of variance at any time. **Figures 6A,C** are typical Lowry plots for C_urine_ at 6 and 10 h showing the most influential parameters in the absence of ethanol and therefore, no inhibition of *m*-xylene metabolism.

However, in the presence of inhibition of *m*-xylene metabolism by ethanol MPY became the most dominant parameter governing variance in urinary excretion of 3-MHA (**Figure 5B**). The main effect of the most influential parameter, MPY, from 4 to 14 h accounted for 44.6–66.7% of variance (**Figure 5B**). R_urine_ increased in proportion of variance from 6.9 to 22.4% from 4 to 10 h post-exposure. No other individual parameter accounted for greater than 11.1% of variance at any time. The switch in the importance of MPY, from insignificance in the absence of ethanol, to the most dominant parameter in the presence of ethanol is consistent with metabolism being an important process for the removal of *m*-xylene. **Figures 6B,D** show the most influential parameters governing variance of C_urine_ at 6 and 10 h in the presence of inhibitory concentrations of ethanol.

The width of the plume indicates that the amount of variance due to interactions ranged from 6.2 and 0.8% (**Figures 6A,C**) in the absence of inhibition to 17 and 22% with inhibition (**Figures 6B,D**). It is noteworthy that in the presence of inhibition, the proportion of variance due to interactions from MPY, KI and PORALDOSE suggest that these parameters are perhaps interacting with each other. This is particularly pronounced at 10 h post-exposure (**Figure 6D**). This may be expected as the model equations use these parameters to describe metabolism. These results also suggest an interaction between VFAC and BW which together determine the mass of adipose tissue.

### Simulation of Venous Blood *m*-Xylene

**FIGURE 7:**
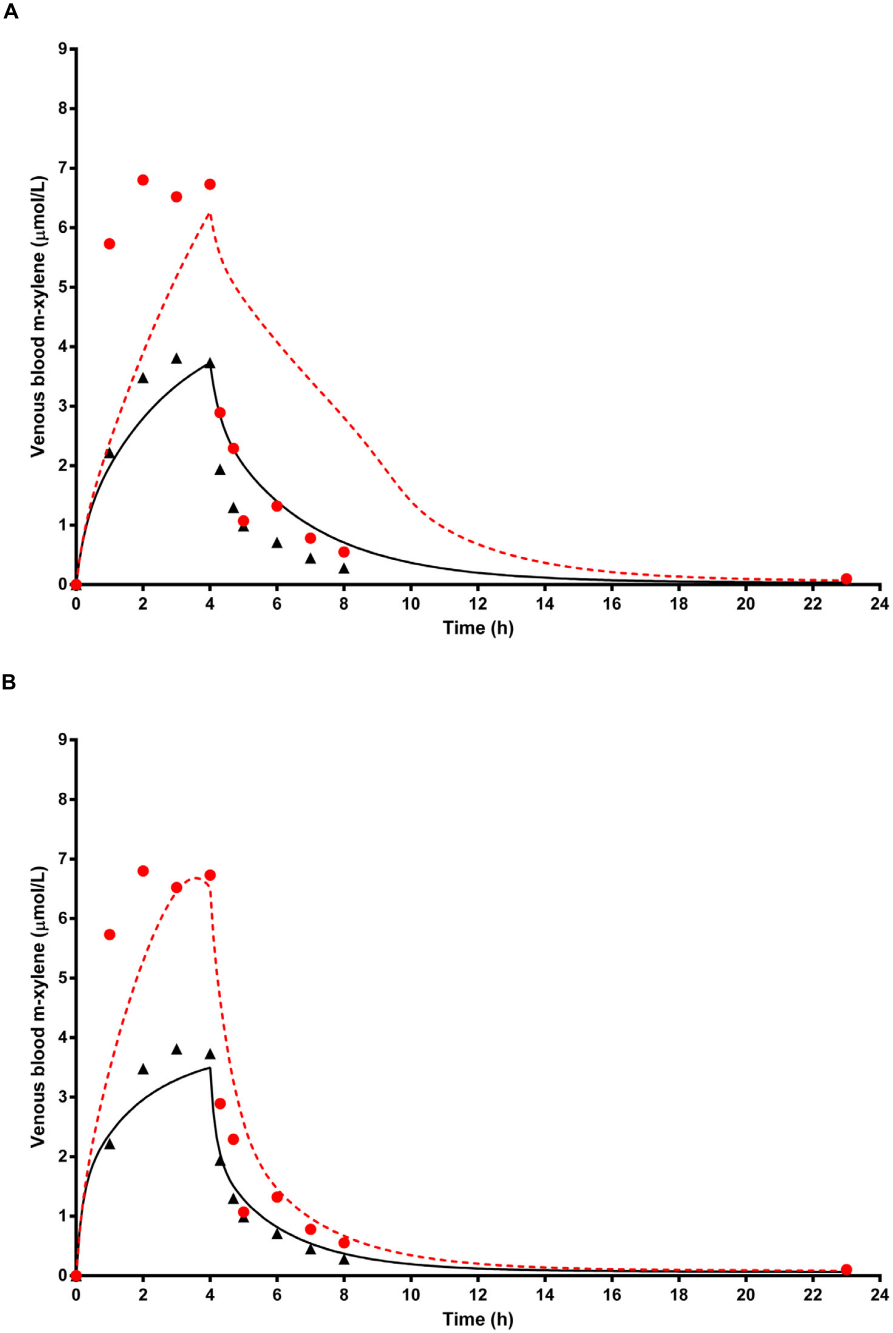
**The full triangle symbols represent measured values for volunteer A without prior ethanol administration, and the solid line is the prediction.** The full red circle symbols represent measured values with prior ethanol administration, and the red broken line is the prediction. **(A)** shows simulations without calibrated, and **(B)** with calibrated most sensitive parameters (MPY and PBAXYL). Parameter abbreviations are listed in **Table 4**.

**Figure 7A** shows the simulated time profiles for CV_xyl_, with and without prior administration of ethanol, for a single subject. This data is representative of the three subjects for whom good measured venous blood data were available. Both predictions were made using the measured parameters for volunteer A (**Table 1**) and a mean value for MPY = 34 mg g^-1^ (Barter et al., 2007). Also, since the value of the inhibitory rate constant, KI obtained from the literature was ineffective (Derr, 1993), a value of 6.57 mg L^-1^ was estimated via the calibration exercise and used for all simulations.

**Figure 7B** shows the same data where the prediction, without prior ethanol administration, was obtained using measured parameters for volunteer A and calibrated values for the most sensitive parameters, MPY = 38.84 mg g^-1^ and PBAXYL = 22.73. Likewise, the prediction, with prior ethanol administration, was obtained using measured parameters for volunteer A and calibrated values for the most sensitive parameters, MPY = 70.35 mg g^-1^ and PBAXYL = 22.04 and KI = 6.57 mg L^-1^.

### Simulation of Exhaled Air *m*-Xylene

**FIGURE 8:**
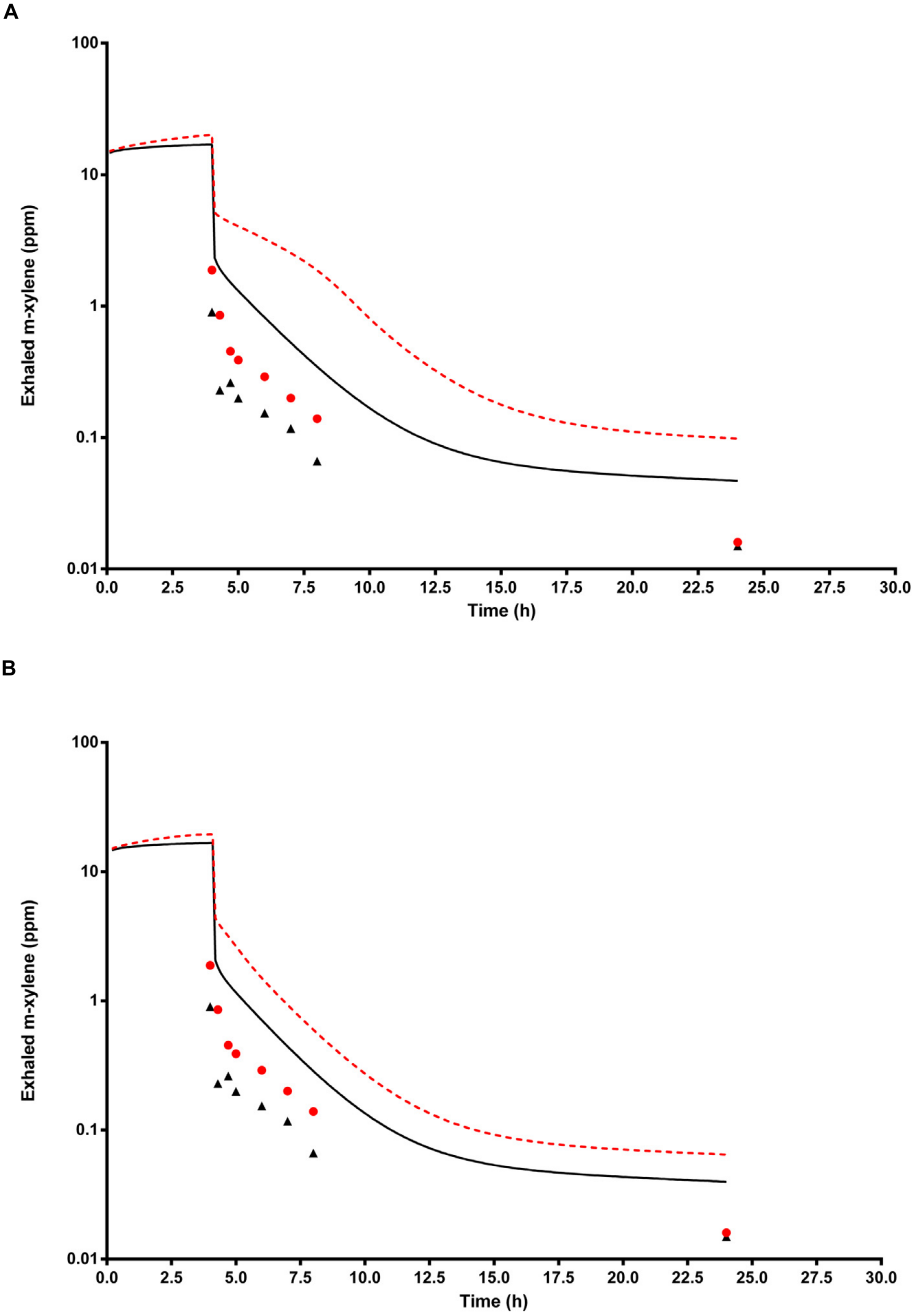
**The full triangle symbols represent measured values for volunteer E, without prior ethanol administration, and the solid line is the prediction.** The full circle symbols represent measured values, with prior ethanol administration, and the broken line is the prediction. **(A)** Shows simulations without calibrated, and **(B)** with calibrated most sensitive parameters (MPY and PBAXYL). Parameter abbreviations are listed in **Table 4**.

**Figure 8A** shows the simulated time profiles for CX_ppm_, with and without prior administration of ethanol, for a single subject. This data is representative of all eight subjects. Both predictions were made using the measured parameters for volunteer E (**Table 1**) and a mean value for MPY = 34 mg g^-1^.

**Figure 8B** shows the same data where the prediction, without prior ethanol administration, was obtained using measured parameters for volunteer E and a calibrated value for the most sensitive parameter, MPY = 60.96 mg g^-1^. Likewise, the prediction, with prior ethanol administration, was obtained using measured parameters for volunteer E and the calibrated value for the most sensitive parameter, MPY = 65.66 mg g^-1^.

### Simulation of Urinary 3-Methylhippuric Acid

**FIGURE 9:**
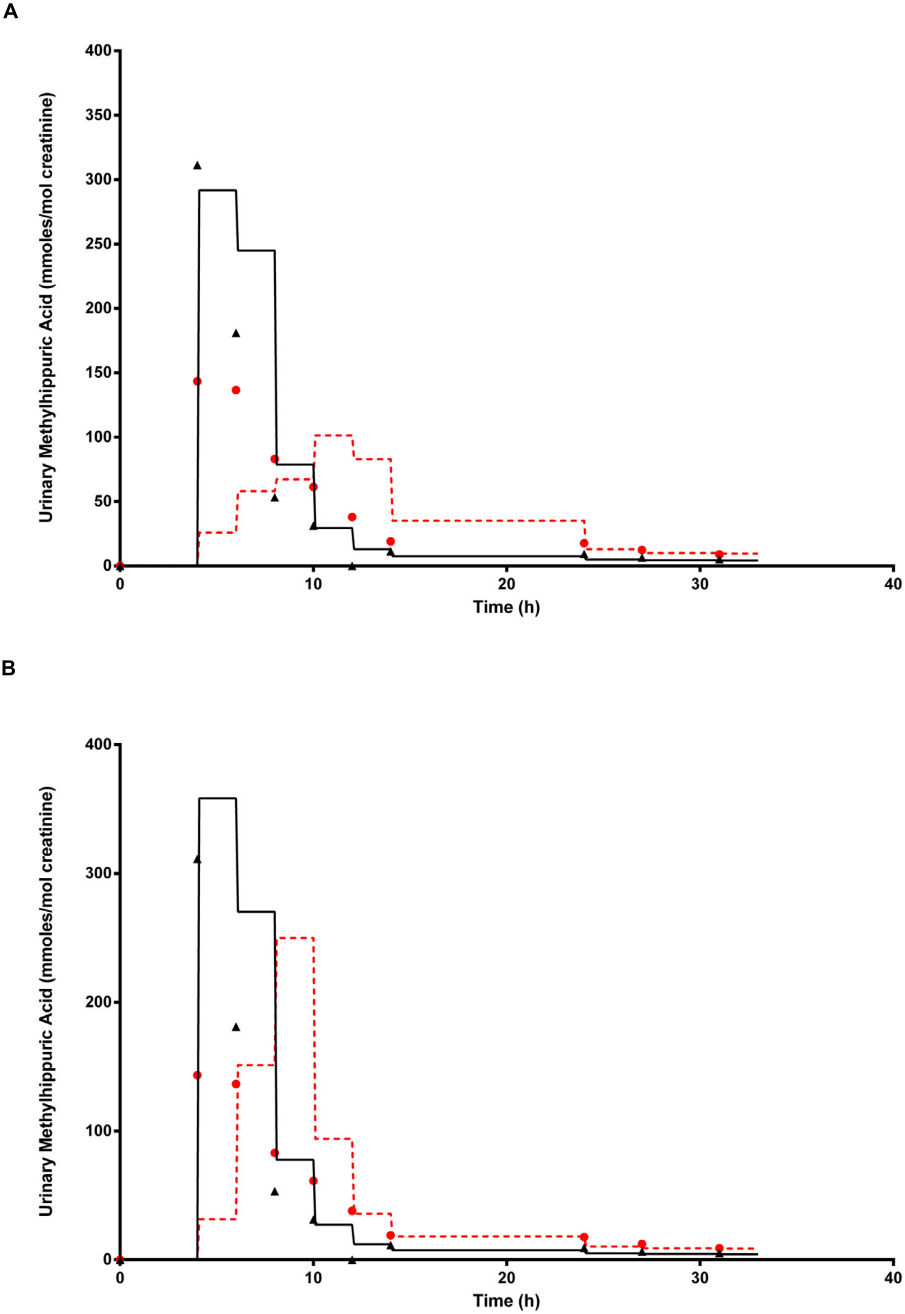
**The full triangle symbols represent measured values for volunteer H, without prior ethanol administration, and the solid line is the prediction.** The full circle symbols represent measured values, with prior ethanol administration, and the broken line is the prediction. **(A)** Shows simulations without calibrated, and **(B)** with the calibrated most sensitive parameter (MPY). Parameter abbreviations are listed in **Table 4**.

**Figure 9A** shows the simulated time profiles for C_urine_, with and without prior administration of ethanol, for a single subject. This data is representative of all eight subjects. The step-like shape of the simulations is the result of the imitation of micturition where the bladder is assumed to fill with urine at a constant (but adjustable) rate and empty at discrete time intervals (when the volume of urine reduces to zero). This enables comparison between model predictions and experimental observations with timed sampling in human volunteer studies (Franks et al., 2006; McNally et al., 2012). Both predictions were made using the measured parameters for volunteer H (**Table 1**) and the mean value for MPY = 34 mg g^-1^ and KI = 6.57 mg L^-1^.

**Figure 9B** shows the same data where the prediction, without prior ethanol administration, was obtained using measured parameters for volunteer H and a calibrated value for the most sensitive parameter, MPY = 43.84 mg g^-1^. Likewise, the prediction with prior ethanol administration, was obtained using measured parameters for volunteer H and the calibrated value for the most sensitive parameters, MPY = 54.12 mg g^-1^ and KI = 6.57 mg L^-1^.

## Discussion

In this study, global SA was used during the model development phase to identify those parameters which had the most significant impact on variability of CV_xyl_, CX_ppm_, and C_urine_. This information informed the selection of parameters for estimation/calibration by fitting to measured BM data in a Bayesian framework using MCMC simulation. This approach ensured that *a priori* knowledge in the form of prior distributions was ascribed only to those parameters that were identified as having the greatest impact on variability. This is an efficient approach which helps reduce computational cost, i.e., simulation times were significantly reduced as reported previously (McNally et al., 2012).

The SA also indicated that adipose mass (VFAC) did not account for greater than 15.4% and respiratory rate (QPMC) 19.2%, of variance for CV_xyl_, CX_ppm_, or C_urine_ at any time. Therefore, calculated values, adjusted to body weight, for both parameters would have been adequate. An initial theoretical modeling exercise during the design phase of the human study may have provided enough confidence to avoid measuring those parameters for each volunteer. This could have represented savings in time and cost.

The most dominant parameter governing variance during the uptake and distribution phase of both CV_xyl_ and CX_ppm_, in the presence or absence of inhibition of *m*-xylene metabolism by ethanol was PBAXYL. PBAXYL is an indication of the solubility of *m*-xylene in human blood which is consistent with a parameter that is important in determining availability of *m*-xylene at the site of metabolism in the liver. With the exception of CX_ppm_ in the absence of inhibition, MPY was the most dominant parameter during the elimination phase of CV_xyl_ and CX_ppm_, which is also consistent with a parameter that determines the rate of metabolism. In the presence of ethanol the proportion of variance governed by PBAXYL and MPY increased for both parameters during the latter elimination phase. This again is consistent with parameters that are important in determining elimination of *m*-xylene by exhalation when metabolism is inhibited.

Calibration of PBAXYL and MPY using Bayesian inference predicted values that were biologically plausible. For all three outputs, CV_xyl_, CX_ppm_, and C_urine_ the MPY values were within the 95% confidence limit of the mean (Barter et al., 2007) as were the PBAXYL values (McNally et al., 2012). It should be noted that the variability in calibrated values for MPY are not necessarily suggesting that microsomal protein yield changes dramatically in the same individual from one study to the next – even when separated by a month. It is a measure of the magnitude of the contribution to variance by that parameter.

The binary chemical PBPK model, with a mechanism of competitive inhibition of hepatic *m*-xylene metabolism, provided satisfactory predictions for CV_xyl_ and C_urine_ although the simulations of CX_ppm_ were over-predicted. It is plausible that the measured exhaled concentrations of *m*-xylene were lower than expected due to the difficulty in sampling the last 150 mL of exhaled air assumed to be in equilibrium with pulmonary arterial blood. In addition, the deviations between model predictions and data for end-exhaled *m*-xylene may be similar to the two possible explanations proposed by Jonsson et al. (2001). The first is that the assumption of instant equilibrium between alveolar air and arterial blood is not valid and the second that model parameters are constant over time at a given workload.

The delay in reaching equilibrium between alveolar air and arterial blood concentrations of several volatiles has been observed in experimentally determined inhalation uptake studies in rats (Johanson and Filser, 1992) and humans (Jonsson and Johanson, 2001a). Further, exhaled concentrations of mercury vapor were best predicted as originating from an additional “respiratory depot” compartment in the respiratory tract (Jonsson et al., 1999). Therefore, the lower measured, end-exhaled concentrations of *m*-xylene may have originated primarily from an upper respiratory tract depot with a much smaller contribution from arterial blood.

Variability in measured end-exhaled concentrations of *m*-xylene (data not shown) may also be partly explained by the variability in blood:air PCs for *m*-xylene. Blood:air PCs for several volatiles have been shown to increase after a meal (perhaps due to variations in the blood lipids; Fiserova-Bergerova et al., 1980) and may explain intra-individual variability in elimination by exhalation and the latter process is inversely proportional to the blood:air PC (Jonsson et al., 2001).

The binary model provided reasonable predictions for the urinary excretion of 3-MHA. The expected delay in the appearance of 3-MHA during inhibition of *m*-xylene metabolism was predicted. The marked switch from insignificant to significant contribution to variance in urinary excretion of 3-MHA by MPY is noteworthy. This is consistent with the importance of metabolism, without which 3-MHA could not be produced. R_urine_ was the next most important parameter after MPY, which is consistent with a process that governs the rate of appearance of a metabolite in the urine.

In summary, a binary chemical PBPK model for ethanol and *m*-xylene co-exposure, with an assumption of competitive inhibition of *m*-xylene metabolism, was used to analyze BM data from a controlled human exposure study. Global SA provided quantitative descriptions of the contributions to variance in the model outputs, CV_xyl_, CX_ppm_, and C_urine_. The identification of the most important parameters governing variance can be used to reduce the computational cost of stochastic simulations. Further, the use of this approach to PBPK model development and analysis may be used to design efficient and cost effective experimental studies.

## Conflict of Interest Statement

The authors declare that the research was conducted in the absence of any commercial or financial relationships that could be construed as a potential conflict of interest.

## Footnotes

1 http://xnet.hsl.gov.uk/popgen

2 http://www.acslx.com/

3 http://lowryplot.appspot.com/

4 www.graphpad.com

5 The ranges of cumulative contribution to variance by the three most influential parameters within a specified time period were obtained by using a spreadsheet to sort the parameter main effects from largest to smallest for each time point shown in **Figures 1, 3 and 5**, and then calculating the sum total.

6 The time trends in the main effect plots appear unusual compared with equivalent plots for exhaled breath and blood and two features are worthy of further explanation. The ‘blocky’ structure on the main effect plots results from the sensitivity analysis results only changing after a micturition event which were simulated every 2 h. Secondly, the increase in importance of PBAXYL at 10 h, and to a lesser extent body mass (BW) and VFAC are not intuitive. There was a very rapid decrease in total variance between 4 and 6 h and a further very large reduction between 6 and 8 h. Plots of the main effect variances (as opposed to the sensitivity indices plotted here) show the main effect variances of each parameter decrease over the period of the simulations, however, the two parameters related to the dilution of 3-MHA in urine decreased at a slower rate compared with other parameters and thus accounted for a greater proportion of the variance in the 6–8 period.
